# From Microscale Interactions to Macroscale Patterns in Copepod–Crinoid Symbiosis

**DOI:** 10.3390/ani14060877

**Published:** 2024-03-13

**Authors:** Oksana A. Korzhavina, Natalia V. Gubareva, Andrey V. Kitashov, Temir A. Britayev, Viatcheslav N. Ivanenko

**Affiliations:** 1 Department of Invertebrate Zoology, Biological Faculty, Lomonosov Moscow State University, Moscow 119991, Russia; korzhavina@mail.bio.msu.ru (O.A.K.);; 2 Faculty of Biology, Shenzhen MSU-BIT University, Shenzhen 518115, China; 3 A.N. Severtsov Institute of Ecology and Evolution, Russian Academy of Science, Moscow 129164, Russia; britayev@yandex.ru

**Keywords:** copepod associations, symbiotic relationships, crinoids, Comatulida, marine biodiversity, marine ecology, marine invertebrates, host-symbiont interactions, marine parasitology

## Abstract

**Simple Summary:**

Crinoids, a class of echinoderms, engage in diverse symbiotic relationships with copepod crustaceans, but our understanding of these interactions remains limited. A review of the literature reveals 163 recorded instances involving 39 copepod species in 6 families associated with 33 species of Comatulida. These associations span 5 of the 12 ecoregions of the World Ocean, with the highest diversity of both symbionts and hosts being noted in the Central and Western Indo-Pacific. Many fewer copepod–crinoid associations have been documented in the Atlantic. Most of these copepods are ectosymbionts, with some instances of endosymbiosis. The genera *Collocheres* Canu, 1893 and *Pseudanthessius* Claus, 1889 are prominent among them, and the host family Comatulidae exhibits the most diverse range of copepod associations. Current records cover only 5% of the potential crinoid host diversity, highlighting the need for further research.

**Abstract:**

Crinoids (Echinodermata) exhibit unique morphological and behavioral characteristics that facilitate a wide range of symbiotic relationships with diverse organisms. Our comprehension of their interactions with microscopic copepod crustaceans is, however, still in a nascent and fragmented state. Here, we review and discuss the 166 literature records to date in which a total of 39 copepod species in 6 families have been reported in association with 33 species of the crinoid order Comatulida. Many of these associations have been reported just once. The respective localities cover 5 of the World Ocean’s 12 ecoregions, with a notable concentration of both host and symbiont diversity in the Central and Western Indo-Pacific. In contrast, the documentation of copepod–crinoid associations in the Atlantic appears markedly limited. Copepods have been found predominantly in ectosymbiotic relationships with crinoids, with a lower incidence of endosymbiosis. Copepods of the genera *Collocheres* Canu, 1893 and *Pseudanthessius* Claus, 1889 are particularly prominent in the list, and the comatulid family Comatulidae displays the most diverse assortment of copepod associations. The current scope of knowledge encompasses a mere 5% of the potential crinoid host diversity, underscoring the need for more extensive research in this area.

## 1. Introduction

The echinoderm class Crinoidea is a diverse and enduring clade with a fossil record stretching back nearly half a billion years [1]. Its modern-day diversity is predominantly found within the order Comatulida, which currently comprises 671 species [2,3]. These crinoids, characterized by limited motility, rudimentary self-cleaning mechanisms, and an absence of saponin secretions, provide a substrate suitable for a variety of vertebrate and invertebrate organisms [4,5]. While direct predation on crinoids is uncommon, their anatomical design—comprising mobile arms, pinnules, and cirri—and their unique filter-feeding method, whereby food particles travel conspicuously along open ambulacral grooves, is conducive to a plethora of symbionts [5,6,7]. The symbiotic taxa often found in association with crinoids include copepod, decapod and thecostracan crustaceans, ophiuroids, fish gastropods, myzostomes, polychaetes, etc. [8,9,10,11,12]. 

In the history of marine biology, a sustained interest in the relationships between crinoids and their symbiotic partners has long been evident. Besides studies devoted to single taxonomic groups, investigations of the entire crinoid symbiont community have been conducted in various coastal ecosystems around the world, notably in the Bay of Bengal [13], the Red Sea [9], the Marshall Islands [14], the Maldives Archipelago [15], Hong Kong [16], the Great Barrier Reef [6], Taiwan [17], New Guinea [7], Japan [18], South Africa [19], Vietnam [5,20] and North Sulawesi [21]. Such investigations have consistently emphasized the prevalence of specialized fauna involved in symbiotic associations with crinoids. However, because of doubts about the accuracy of species identification, prudence is necessary in interpreting these findings. Furthermore, a conspicuous gap in data persists regarding the undoubtedly diverse and ecologically important, yet insufficiently studied, microscopic symbionts of crinoids, such as myzostomids and copepods [22,23,24].

Copepods play a significant, though still insufficiently explored, role in a wide range of ecological interactions within marine ecosystems [25,26,27,28]. Their symbiotic relationships with various echinoderm species taxa in diverse marine environments underscore their remarkable ecological adaptability and highlights the intricate network of biotic interactions in which copepods take part. On echinoderms, copepods of various families have been observed in association with Crinoidea (feather stars), Asteroidea (sea stars), Echinoidea (sea urchins), Holothuroidea (sea cucumbers), and Ophiuroidea (brittle stars) [29,30,31,32,33,34,35]. The investigation of these interactions not only reveals the ecological significance of copepods but also contributes to a deeper understanding of the evolutionary mechanisms that underlie symbiosis within marine ecosystems [28,31].

This study is a component of a broader project aimed at elucidating patterns and assessing the depth of understanding pertaining to copepod symbionts found in association with various invertebrates, with a particular focus on echinoderms, sponges, and corals [36,37,38,39,40]. Through an exhaustive analysis and synthesis, we aspire to offer a holistic view of these relationships, focusing on their ecological, evolutionary, and taxonomic dimensions, thereby enhancing our comprehensive understanding of marine symbiotic systems and the pivotal roles that copepods play in them.

## 2. Materials and Methods

We developed a comprehensive Microsoft Access database to meticulously analyze the symbiotic interactions between copepods and crinoids. This database comprises four tables—‘Hosts’, ‘Symbionts’, ‘Sites’, and ‘Publications’—which collectively merge into a comprehensive ‘Literature Records’ table (Table 1, Table A1 and Table S1). Adhering to the approaches set by the World Register of Marine Species [41], this database provides a thorough record of the taxonomic classification of both the hosts and symbionts. It also incorporates a broad spectrum of meticulously completed data, including detailed information on symbiotic relationships, geographical locations, sampling depths, and timestamps in accordance with the Darwin Core data standards [42] (Table S2 for further details). To ensure consistency and accuracy in taxonomic nomenclature in the database, we employed the ‘Taxon Match’ tool in WoRMS, crucial in handling the evolving taxonomy of the host crinoids. The classification of oceanic ecoregions followed the classification by Spalding [43]. We do not endorse the recently proposed changes in the taxonomic status changes to the order Poecilostomatoida, as we believe this topic warrants further investigation [44,45]. Spatial data were processed by extracting the geographical coordinates for each sampling location from the original literature and subsequently georeferencing them. Visualization of the geographic data points was achieved using the digital mapping platforms Google Maps and RStudio Version 1.2. To visualize the data and generate plots, we first employed Rstudio version 1.2.5001, harnessing the capabilities of various packages such as *tidyverse* [46], *dplyr* [47], *ggplot2* [48], *ggExtra* [49], *ggpubr* [50], *gridExtra* [51], *magrittr* [52], *maps* [53], *stringr* [54], and *RcolorBrewer* [55], and then crafted the final graphical representations using Adobe Photoshop CC.

## 3. Results and Discussion

### 3.1. The History of Research

**Figure 1:**
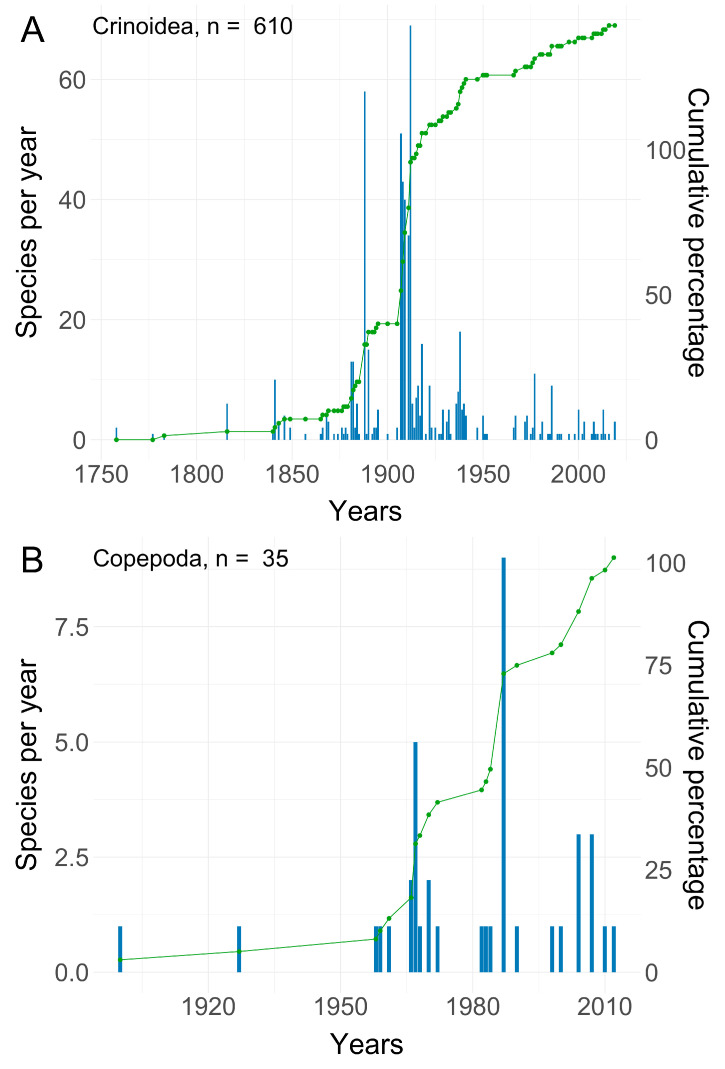
Numbers of new species and cumulative percentage (green line) of known species of (**A**) crinoids and (**B**) symbiotic copepods associated with them and described in publications over time. Based on the WoRMS database [41].

Over the past century, the study of copepod symbionts associated with crinoids has resulted in the publication of 24 scientific articles (Table 1) documenting symbiotic interactions between copepods and crinoids (Figure 1). The gathering of taxonomic knowledge about copepod symbionts has been remarkably slower than the trajectory of taxonomic coverage might suggest. This discrepancy can be attributed to the inherent challenges associated with collecting microscopic symbionts residing within galls and internal organs in addition to those inhabiting the external surfaces of crinoids.

The advancement of SCUBA diving techniques for sampling shallow-water environments, which began in the 1950s, facilitated the gathering of 157 records documenting the presence of copepods living in association with shallow-water crinoids. Over the past decade, however, there has been a noticeable decline in such research activity. Few works have focused on morphological descriptions, with some providing brief comments on the zoogeographical aspects and relationships between copepods and their hosts. This trend underscores the existence of numerous unexplored facets in the symbiotic relationship between copepods and crinoids, particularly concerning the nature of the symbiosis and its implications for both partners.

### 3.2. Sampling Methods and Challenges

The choice of methodology is inevitably linked to the type of symbiotic interaction found. Specimens isolated via washing are consistently categorized as ectosymbiotic, with descriptions typically lacking details of the precise location on the host. On the contrary, specimens isolated via copepod dissection are invariably classified as endosymbiotic, and precise information about their location in the host is typically given, e.g., within the intestinal tract (Grainger 1950 [59,60,61,62,70], within the galls [64], or the coelom [59]. The significant methodological influence on the types of copepods detected suggests that the spectrum of endosymbiotic copepods associated with crinoids remains incompletely explored [10].

In the study of copepod–crinoid symbiosis, dissection has been relatively infrequent, limited to 11 instances marked as endosymbionts (Table A1 and Table S1). The prevailing methodology for copepod identification involves the use of a 5% ethanol solution to wash the crinoid hosts. This approach, although efficient and capable of recovering a significant diversity of microsymbionts, presents challenges when conducting quantitative assessments. This method has nonetheless provided the majority (155) of published observations related to copepods on crinoids, powerfully shaping our comprehension of copepod–host relationships. Consequently, further research is imperative to ascertain the precise localization of the majority of ectosymbionts.

Research into copepod–crinoid symbioses faces significant challenges, particularly the difficulty of collecting loosely attached external symbionts from deep-sea crinoids. This problem is compounded by the current state of knowledge about microscopic copepods residing in or on crinoids and the lack of an integrative approach to their systematics, especially regarding the application of molecular methodologies [36,78]. Much of the existing data are derived from faunistic or exploratory studies, which, while inevitable in the initial phase of study of researching any taxonomic group, constrain the breadth and depth of understanding. 

### 3.3. Diversity and Taxonomy of Symbiotic Copepods

**Figure 2:**
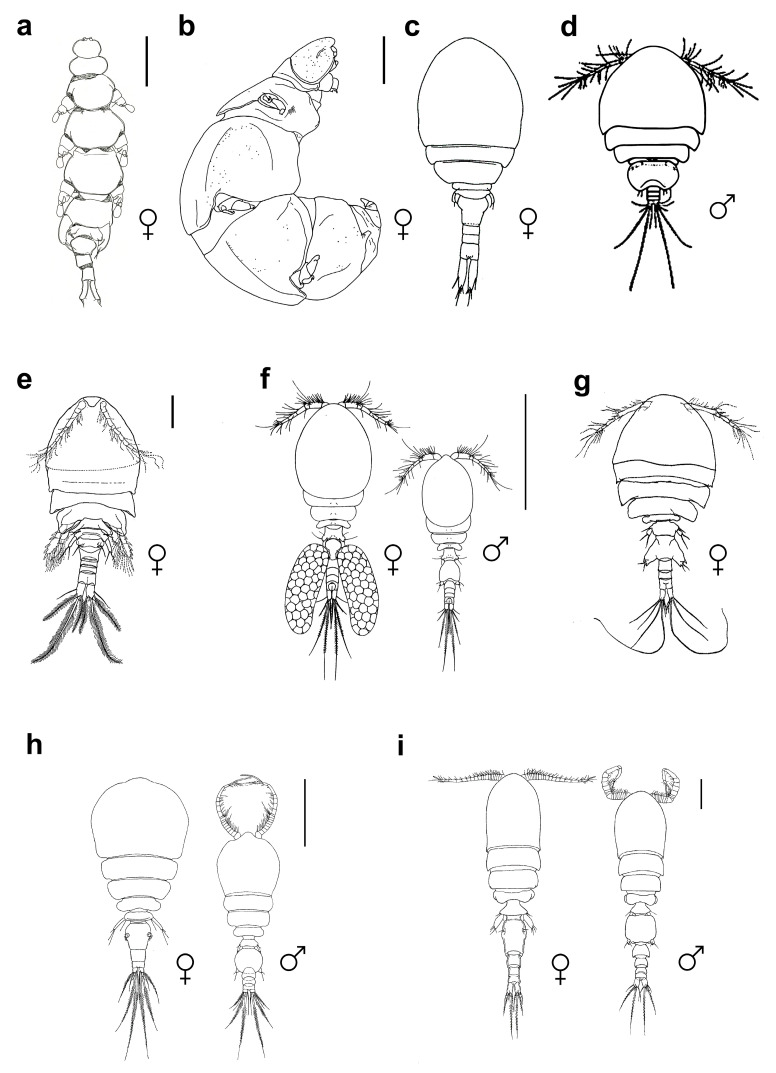
Habitus of copepod crustaceans living on crinoids. (**a**) *Enterognathus inabai*, dorsal view, scale bar 1 mm; (**b**) *Parenterognathus troglodytes*, lateral view, scale bar 0.5 mm; (**c**) *Critomolgus fishelsoni*, dorsal view, scale bar 0.5 mm; (**d**) *Doridicola patulus*, dorsal view; (**e**) *Kelleria gradata*, ventral view, 0.2 mm; (**f**) *Pseudanthessius comanthi*, both sexes are shown, dorsal view, 0.5 mm; (**g**) *Scambicornus pillaii*, dorsal view, 0.1 mm; (**h**) *Asterocheres crinoidicola*, both sexes are shown, dorsal view, 0.3 mm; (**i**) *Collocheres brevipes*, both sexes are shown, dorsal view, 0.1 mm; ((**a**,**b**) Cyclopoida, (**c**–**g**) Poecilostomatoida, (**h**,**i**) Siphonostomatoida). After [65] (**a**), [64] (**b**), [73] (**c**,**e**), [77] (**d**), [53] (**f**), [75] (**g**), [10] (**h**), [66] (**i**).

Our literature review uncovered 166 instances involving 39 copepod species representing 6 families in association with 33 species of the crinoid order Comatulida. Intimate symbiotic associations have been identified among members of three orders of copepods—Cyclopoida, Poecilostomatoida, and Siphonostomatoida—and various comatulids (Table 2 and Table 3 and Figure 2), constituting more than 5% of the known diversity within the order. 

The orders Poecilostomatoida and Siphonostomatoida display parallel trends in their frequency of occurrence and the spectrum of crinoid taxa that they are associated with (Table 2). A particularly broad spectrum of families and genera of Poecilostomatoida are involved with crinoids, with 82 instances of such associations having been reported to date. This points towards the need for more in-depth exploration of their symbiotic links with crinoids, which may turn out to be more elaborate than those of other copepod orders. The order Siphonostomatoida, including 19 species with known crinoid associations, also shows a heightened level of specialization.

Among the 95 families in the order Cyclopoida, only members of the Enterognathidae have been found in crinoids. Four of the seven known species in this family have been found to be associated with a total of seven species of feather star (Table 2). The number of literature records has varied among these four (mean 2.75, SE 0.85), and half of them (two species) have only a single known host.

The order Poecilostomatoida is predominantly represented by seven species of Pseudanthessiidae, which are associated with a total of 15 species of feather star (Table 2). This family is notable for its high number of literature records per species (mean 13.57, SE 5.03), and two of them (*Pseudanthessius rostellatus* Humes, Ho, 1970 and *P. planus* Kim, 2007) appear at present to associated with a single host species (Table A1).

The order Siphonostomatoida is represented by only one family, Asterocheridae; 19 of its species are associated with 17 species of crinoids (Table 2). The number of literature records per species (mean 3.8, SE 1.17) varies considerably. A substantial proportion of crinoid-associated asterocherids (63.16%) have only a single known host species, suggesting a trend towards species-specific symbiosis. 

Despite the diversity of invertebrate-associated copepods within the large families Asterocheridae, Rhynchomolgidae, and Pseudanthessiidae, only a small fraction of each are found to be in association with crinoids—7% of 271 species, 2.5% of 270 species, and 5% of 61 species, respectively. These data highlight the potential for multiple evolutionary transitions between host invertebrates (host switching), as well as the insufficient investigation into the diversity and phylogeny of these symbiotic copepod families.

### 3.4. Specialization in Copepod–Crinoid Symbiosis

The examination of morphological adaptations in copepods, particularly those engaged in endosymbiotic relationships with crinoids, reveals significant deviations from a typical crustacean morphology (Figure 2). Among the present copepods, this phenomenon is especially notable in the cyclopoid family Enterognathidae, which is predominantly associated with crinoids and is represented by such genera as *Enterognathus* Giesbrecht, 1900 (Figure 2a) and *Parenterognathus* Ohtsuka, Kitazawa & Boxshall, 2010 (Figure 2b). Descriptions of various endosymbiotic or gall-inducing copepods have shown that endosymbiosis leads to considerable morphological changes in the copepods (Figure 2). In the case of Enterognathidae, these include a swollen, vermiform body with reduced segmentation and sclerotization, obscured demarcation between the prosome and urosome, and the diminution or complete absence of antennae and maxillipeds. These modifications of Enterognathidae are markedly more pronounced compared to those of other crinoid-dwelling copepods, but are nonetheless an extreme form of those observed in members of the family Lamippidae (Cyclopoida), obligate symbionts of octocorals [38,39].

The order Comatulida encompasses a diverse array of confirmed crinoid hosts for copepods, including 8 families, 21 genera, and 33 species, as detailed in Table 3 and Table S1. The family Comatulidae is a prominent host group for symbiotic copepods. Only 16% of its 102 species are known to serve as hosts for copepods; nonetheless, feather stars of this family are involved in half of all recorded copepod findings from crinoids (81 out of 166), with half of all known crinoid-symbiont copepod species (20 out of 40) as their partners (Table 3). The family Antedonidae, however, despite its total diversity (151 species), boasts a very small number of confirmed symbiotic relationships with copepods. These apparent differences among crinoid families in the prevalence of symbiotic relationships with copepods might reflect an uneven distribution of research efforts, but they also underscore the potential influence of complex ecological and evolutionary factors, including selectivity, that may warrant further investigation.

**Figure 3:**
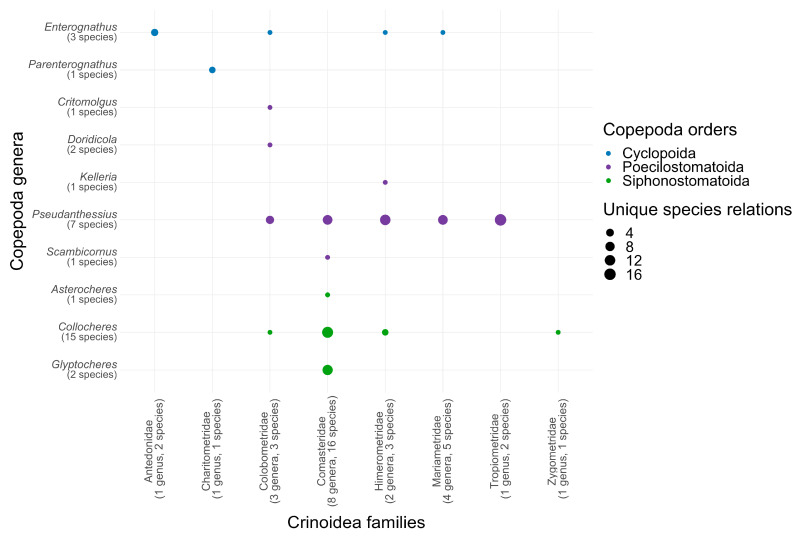
Number of records per association of symbiotic copepod genera with crinoid families. The size of each oval mark denotes the number of records.

The degree of host specificity exhibited by various copepod species in their interactions with crinoids ranges from highly specific to more flexible (Figure 3). There are 20 species of single-species-specific symbionts, 61% of the total, as opposed to 13 species (39%) associated with a variety of hosts. For example, *Pseudanthessius major* Stock, 1967 and *P. minor* Stock, 1967 have multiple host species (eight and four, respectively) and host genera (seven and four, respectively), while *Collocheres uncinatus* Stock, 1966 is associated with three host families: Colobometridae, Comatulidae, and Himerometridae. The different degrees of host specificity are likely indicative of diverse trajectories in the evolutionary ecology of these copepods.

A restricted number of host species is typically linked to the evolution of specific adaptive traits. This is exemplified by *Pseudanthessius angularis* Humes, Ho, 1970, *P. comanthi* Humes, 1972 (Figure 2f), and *P. madrasensis* Reddiah, 1968, which are characterized by the development of prominent egg sacs in females, a feature commonly observed in symbiotic copepod species that distinguishes them from such species as *P. major* and *P. minor*. Additionally, *Enterognathus comatulae* Giesbrecht, 1900 and *E. lateripes* Stock, 1966 display significant morphological adaptations, including a vermiform body with inflated, rounded body segments and fringed swimming appendages.

The single-host-specific *Parenterognathus troglodytes* Ohtsuka, Kitazawa, Box-shall, 2010 exhibits a more pronounced degree of body modification than its counterparts. Distinct adaptations are also evident in *P. planus* Kim I.H., 2007 and *P. rostellatus* Humes & Ho, 1970, including broader and rounder thoracic segments in the former and an abundance of long setae on the antennae and urosome in the latter. *Kelleria gradata* Stock, 1967 (Figure 2e), another species-specific symbiont, has elongated setae, particularly on the swimming legs, and expanded thoracic segments, a feature also observed in *Critomolgus fishelsoni* (Stock, 1967) (Figure 2c) and *Doridicola patulus* (Humes, 1959) (Figure 2d).

Any attempt at a comprehensive comparative analysis of these copepods’ morphological adaptions will face many challenges, primarily due to variations in the depth of sampling, taxonomic precision, and accuracy in species identification. Moreover, our understanding of host specificity is constrained by the fragmentary nature of the available data. This is true for the majority of copepods found associated with invertebrates, as noted by Ivanenko [36].

### 3.5. Distribution of Crinoid-Associated Copepods

**Figure 4:**
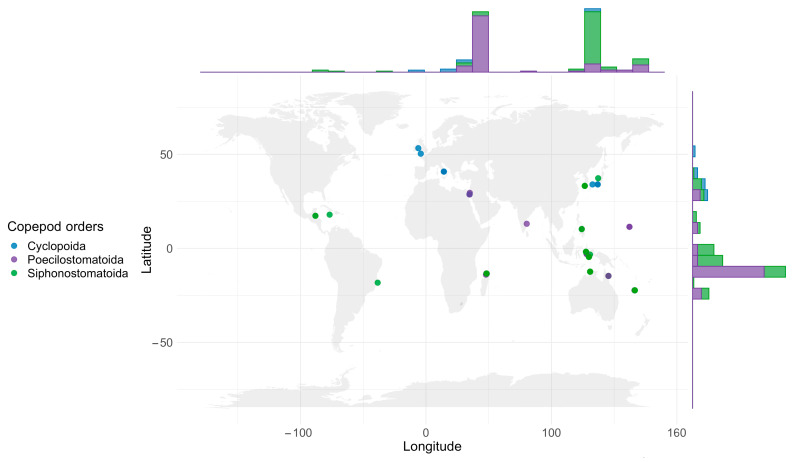
Distribution of the copepods associated with crinoids in the World Ocean. The marginal histogram illustrates the latitudinal and longitudinal distribution of the reports of copepods.

Copepods engaged in symbiosis with crinoids are distributed across a wide range of ecosystems, extending from tropical to temperate latitudes in both the Western and Eastern hemispheres (Figure 4). Observations have been concentrated predominantly in the tropical zones of both hemispheres, with comparatively few data originating from the temperate latitudes. Symbiotic interactions of this sort have been documented in 5 of the 12 marine ecoregions, delineated by Spalding [43], with the Central and Western Indo-Pacific being the richest in terms of taxonomic diversity (Table 4). 

Taking Figure 4 at face value, the biodiversity hotspots for these organisms include Madagascar, Australia, and the Indo-West Pacific archipelagos, with notable concentrations in northern Madagascar, the Moluccas of Indonesia, and New Caledonia. These data were largely obtained from fieldwork conducted by Prof. Arthur Humes [79]. Several species of symbiotic copepods, namely *Collocheres prionotus* Humes, 1990, *C. uncinatus*, *Pseudanthessius madrasensis* Reddiah, 1968, and *P. major*, alongside host crinoids such as *Capillaster multiradiatus* (Linnaeus, 1758) and *Stephanometra indica* (Smith, 1876), show a pan-Indo-Pacific distribution (Table A1). The heightened diversity observed in regions like Indonesia and Madagascar is a result of intensive sampling efforts there, suggesting that further research could reveal additional species elsewhere. The temperate North Pacific and North Atlantic regions are markedly less studied in this respect, with only rare findings of the endoparasitic *Enterognathus comatulae* [59,62]. Species such as *Collocheres comanthiphilus*, *Pseudanthessius major*, and *Glyptocheres extrusus* Humes, 1987 are of particular interest due to their widespread distribution among multiple Indo-Pacific sites [22,58,73]. Additionally, the cosmopolitan presence of the scarcely documented genus *Enterognathus*, encompassing the northeast Atlantic, the Red Sea, and Japanese waters, presents a compelling case for further investigation (Table A1).

The paucity of data in certain geographical areas can be attributed to either the absence of copepod populations or to the lack of extensive research in these locales. The distribution pattern of existing data may reflect the habitat preferences inherent to copepod species, as well as highlight areas of specific interest within the research community. This pattern underscores the critical need for enhanced research efforts in underexplored regions to attain a holistic understanding of the global distribution patterns of these marine symbionts.

### 3.6. Bathymetric Distribution

An analysis of the depth data (Table A1) may offer an insight into the habitats in which symbiotic partnerships between copepods and crinoids have been established. The high concentration of relevant findings within the upper 50 m or so (mostly the upper 25 m), in contrast to the rarity of finds in the deep sea, has two possible explanations: a real preference for establishing these symbiotic relationships in shallow water, or a research bias that has focused on sampling in more accessible, shallower waters. 

**Figure 5:**
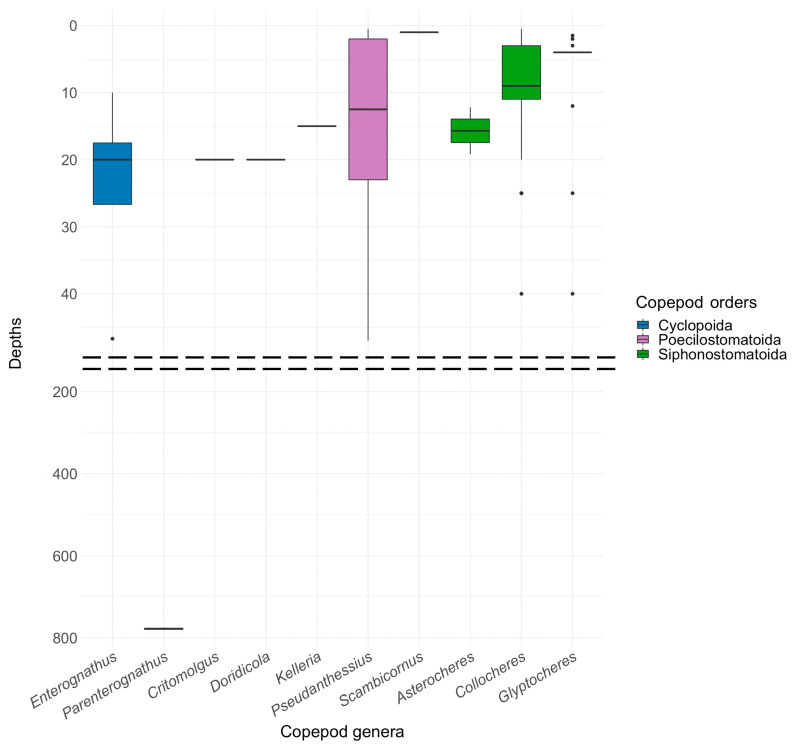
Distribution of symbiotic copepods associated with crinoids by depth. The horizontal line within each box represents the median of the dataset. The box defines an interquartile range from the 25th to the 75th percentile. Vertical lines extending from each box show the minimum and maximum data values. Data points appearing outside of these vertical lines are identified as outliers. Dashed lines demarcate depths not depicted, where no instances of copepod presence on crinoids were recorded.

An obvious connection is observed between the known bathymetric distribution of the associated copepods and the preferred habitats of their host crinoids in the order Comatulida (Figure 5). The shared ecological circumstances in shallower marine zones may have played a crucial role in facilitating, establishing, and maintaining these symbiotic relationships, resulting in a long co-evolutionary relationship. The presence of symbiotic copepods in deeper waters, albeit much less frequently documented, opens avenues for further exploration into the adaptive capabilities and ecological breadth of these symbiotic copepods. The discovery of the copepod *Parenterognathus troglodytes* in a bathyal crinoid shows that the copepods themselves are adaptable to life at depth.

Differences are evident in the bathymetric distribution of symbiotic copepods belonging to different orders (Table A1, Figure 5). The cyclopoids are represented by a single genus that favors relatively shallow waters, mostly around 20 m deep. In contrast, the crinoid-associated poecilostomatoids inhabit a greater range of depths, namely 10 m–40 m and below. While the crinoid-associated siphonostomatoids also occur at various depths, their range is narrower than that of the poecilostomatoids and is focused at around depths of 10 m. Certain copepod genera, such as *Collocheres* and *Enterognathus*, appear to inhabit a very narrow depth range. Outlying data points in Figure 5, particularly for poecilostomatoids, may signal the presence of rare species that prefer significantly deeper waters than most.

## 4. Conclusions

This series of conclusions reached here are only tentative, tempered by the recognition of substantial gaps in the existing body of knowledge. These gaps result from disparities in the depth and scope of studies, variations in taxonomic precision, and inconsistencies in the identification of symbiotic taxa. The exploration of copepod–crinoid symbioses, representing a substantial yet largely uncharted domain of scientific inquiry, faces notable challenges including a prevailing research bias towards macro-symbionts, inherent difficulties in the collection and analysis of micro-symbiont data, and the still nascent stage of research in this field. The enhancement and standardization of research methodologies are imperative and must be specially tailored to the study of marine invertebrates’ relationships with microscopic symbionts. There is a need for more comprehensive and in-depth research focused on microscopic crustaceans, using robust and innovative methodology [27,80].

Current knowledge in the field of copepod–crinoid symbiosis is based on only a fraction—approximately 5%—of the hypothesized diversity of crinoid hosts. The preliminary calculations conducted by us, based on the observed diversity of copepods associated with crinoids and relying solely on morphological studies, suggest that a minimum of 600 species of crinoid-associated copepods remain to be described. The application of molecular methods, which in other taxa had considerable success in revealing species diversity that had previously gone unrecognized at the morphological level, may be expected to exponentially increase these preliminary estimates [36,78]. Such approaches are essential for fully unraveling the complexities and nuances of symbiotic relationships in marine ecosystems. 

## Figures and Tables

**Table 1:** List of references reporting records of copepods, divided by world ocean region and country (for more details see Table A1 and Table S1).

Region	Countries	References
Central Indo-Pacific	Australia	[22]
	Indonesia	[22,32,56]
	Marshall Islands	[57]
	New Caledonia	[22,58]
	Philippines	[22]
Temperate North Atlantic	France	[59]
	Ireland	[60]
	Italy	[61,62]
	United Kingdom	[60]
Temperate North Pacific	Japan	[63,64,65]
	Korea	[66]
Tropical Atlantic	Belize	[10]
	Brazil	[67]
	Jamaica	[68]
Western Indo-Pacific	India	[69]
	Israel	[70,71,72,73,74]
	Madagascar	[55,72,75,76,77]

**Table 2:** The families of Copepoda in relation to Crinoidea *.

Taxa	Total No of Known Copepod Species	No of Species Found on Crinoids	No of Relevant Literature Records	No of Associated Crinoid Families	No of Associated Crinoid Genera	No of Associated Crinoid Species	Mean No Records Per Copepod Host Species ± SE **	Mean No of Host Species Per Copepod Species ± SE	% of Crinoid- Associated Copepod Species with a Single Host Species
Cyclopoida									
Enterognathidae	7	4	11	5	6	7	2.75 ± 0.85	1.75 ± 0.48	50
Poecilostomatoida									
Kelleriidae	19	1	1	1	1	1	1 ± NA ***	1 ± NA	100
Pseudanthessiidae	61	7	77	6	13	14	13.57 ± 5.03	3 ± 0.93	28.57
Rhynchomolgidae	270	3	3	1	2	2	1 ± 0	1 ± 0	100
Synapticolidae	50	1	1	1	1	1	1 ± NA	1 ± NA	100
Siphonostomatoida									
Asterocheridae	271	19	73	5	12	17	3.84 ± 1.17	2 ± NA	63.16
Total	678	35	166	19	35	42			

* WoRMS database [41]. ** SE (standard error) represents the standard error for calculating the mean value. *** NA (not available) indicates the unavailability or inapplicability of data in a set.

**Table 3:** Crinoidea families in relation to copepods *.

Host Taxa	No of Genera	No of Genera Hosting Copepods (% of Total)	No of Species	No of Species Hosting Copepods (% of Total)	No of Records	No of Copepod Species Involved	No of Host Species with			
							1	2	3	4
							Copepod Species			
Comatulida										
Antedonidae	50	1 (2%)	151	2 (1.32%)	5	1	2			
Charitometridae	8	1 (12.5%)	33	1 (3.03%)	2	1	1			
Colobometridae	18	3 (16.67%)	47	3 (6.38%)	11	6	1		2	
Comatulidae	23	8 (34.78%)	102	16 (15.69%)	81	20	7	5	3	1
Himerometridae	5	2 (40%)	39	3 (7.69%)	18	6		2		1
Mariametridae	7	4 (57.14%)	22	5 (22.73%)	24	4	1	3	1	
Tropiometridae	1	1 (100%)	4	2 (50%)	21	1	2			
Zygometridae	2	1 (50%)	10	1 (10%)	1	1	1			
Total	114	21	408	33	163	40	15	10	6	2

* WoRMS database [41].

**Table 4:** Distribution of symbiotic copepods and their crinoid hosts in various ecoregions *.

Region	No of Localities	No of Records	No of Symbiont Orders	No of Symbiont Families	No of Symbiont Genera	No of Symbiont Species	No of Host Families	No of Host Genera	No of Host Species
Central Indo-Pacific	21	74	2	2	3	17	4	9	14
Temperate North Atlantic	4	5	1	1	1	1	1	1	2
Temperate North Pacific	5	11	2	2	3	6	4	4	5
Tropical Atlantic	1	2	1	1	1	1	1	2	2
Western Indo-Pacific	23	71	3	6	7	13	5	11	12

* WoRMS database [41].

## Data Availability

The data presented in this study are openly available at https://doi.org/10.15468/p2dsmy (accessed on 10 March 2024).

## Supplementary Materials

The following supporting information can be downloaded at: https://www.mdpi.com/article/10.3390/ani14060877/s1, Table S1: Crinoids as hosts of copepod crustaceans; Table S2: Description of the dataset with specific information relative to column names, description, units, and attribute type.

## Appendix A

**Table A1:** Copepod crustaceans recorded as being associated with crinoids (see also Table S1. Crinoids as hosts of copepod crustaceans).

Copepod	Host Species: Valid Name and [as in Original Record]	Host Family Abbreviation *	Symbiosis Nature Abbreviation **	Collection Site Abbreviation ***	Depth (m)	Reference
Cyclopoida						
Enterognathidae						
*Enterognathus comatulae* Giesbrecht, 1900	*Antedon bifida* (Pennant, 1777)	A	en	GB, IE		[60]
*Enterognathus comatulae* Giesbrecht, 1900	*Antedon mediterranea* (Lamarck, 1816)	A	en	FR, IT		[59,61,62]
*Enterognathus inabai* Ohtsuka, Shimomura, Kitazawa, 2012	*Lamprometra* sp.	M	en	JP	46.7–46.9	[64]
*Entherognathus lateripes* Stock, 1966	*Decametra chadwicki* (Clark, 1911)	Col	en	IL	20	[71]
*Entherognathus lateripes* Stock, 1966	*Oligometra serripinna* (Carpenter, 1811)	Col	en	IL	20	[71]
*Entherognathus lateripes* Stock, 1966	*Heterometra savignii* (Müller, 1841) [as *Heterometra savignyi* (Müller, 1841)]	H	en	IL	10	[71]
*Parenterognathus troglodytes* Ohtsuka, Kitazawa, Boxshall, 2010	*Glyptometra crassa* (Clark, 1912)	Ch	en	JP	775, 780.8–787.1	[63]
Poecilostomatoida						
Kelleriidae						
*Kelleria gradata* Stock, 1967	*Heterometra savignii* (Müller, 1841) [as *Heterometra savignyi* (Müller, 1841)]	H	ec	IL	15	[72]
Pseudanthessiidae						
*Pseudanthessius angularis* Humes, Ho, 1970	*Stephanometra indica* (Smith, 1876) [as *Stephanometra spicata* (Carpenter, 1881)]	M	ec	MG	1	[75]
*Pseudanthessius angularis* Humes, Ho, 1970	*Anneissia bennetti* (Müller, 1841) [as *Comanthus bennetti* (Müller, 1841)]	M	ec	MG	2, 6	[75]
*Pseudanthessius comanthi* Humes, 1972	*Comanthus wahlbergii* (Müller, 1843)	Com	ec	MH	4, 8	[53]
*Pseudanthessius comanthi* Humes, 1972	*Oxycomanthus bennetti* (Müller, 1841) [as *Comanthus bennetti* (Müller, 1841)]	Com	ec	ID	25	[22]
*Pseudanthessius comanthi* Humes, 1972	*Heterometra savignii* (Müller, 1841) [as *Heterometra savignyi* (Müller, 1841)]	Com	ec	AU, ID, PH	2, 3, 4, 10, 12, 40	[22]
*Pseudanthessius madrasensis* Reddiah, 1968	Comatulida		ec	IN		[69]
*Pseudanthessius madrasensis* Reddiah, 1968	*Tropiometra afra* (Hartlaub, 1890)	T	ec	NC	1.5, 2, 3	[58]
*Pseudanthessius madrasensis* Reddiah, 1968	*Tropiometra carinata* (Lamarck, 1816)	T	ec	MG	0.5, 1, 1.5, 2, 3, 15	[75]
*Pseudanthessius major* Stock, 1967	*Cenometra emendatrix* (Bell, 1892)	Col	ec	MG	10, 20	[72]
*Pseudanthessius major* Stock, 1967	*Heterometra africana* (Clark, 1911)	H	ec	MG	17, 18, 25, 29, 34	[72]
*Pseudanthessius major* Stock, 1967	*Heterometra savignii* (Müller, 1841) [as *Heterometra savignyi* (Müller, 1841)]	H	ec	IL	10, 15	[72]
*Pseudanthessius major* Stock, 1967	*Himerometra robustipinna* (Carpenter, 1881) [as *Himerometra magnipinna* Clark, 1908]	H	ec	NC	1	[58]
*Pseudanthessius major* Stock, 1967	*Dichrometra flagellata* (Müller, 1841) [as *Dichrometra afra* Clark, 1912]	M	ec	MG	1, 2, 6	[72]
*Pseudanthessius major* Stock, 1967	*Lamprometra palmata* (Müller, 1841) [as *Lamprometra klunzingeri* (Hartlaub, 1890)]	M	ec	MG	1, 13	[72]
*Pseudanthessius major* Stock, 1967	*Liparometra* sp.	M	ec	MG	15, 23, 27, 35	[72]
*Pseudanthessius major* Stock, 1967	*Stephanometra indica* (Smith, 1876) [as *Stephanometra spicata* (Carpenter, 1881)]	M	ec	MG, NC	2, 3, 13, 17	[58,72]
*Pseudanthessius minor* Stock, 1967	*Heterometra africana* (Clark, 1911)	H	ec	MG	18	[75]
*Pseudanthessius minor* Stock, 1967	*Dichrometra flagellata* (Müller, 1841) [as *Dichrometra afra* Clark, 1912]	M	ec	MG	2	[75]
*Pseudanthessius minor* Stock, 1967	*Lamprometra palmata* (Müller, 1841) [as *Lamprometra klunzingeri* (Hartlaub, 1890)]	M	ec	IL, MG	0.5, 13	[75]
*Pseudanthessius minor* Stock, 1967	*Liparometra* sp.	M	ec	MG	15, 23, 27, 35	[75]
*Pseudanthessius planus* Kim, 2007	*Himerometra robustipinna* (Carpenter, 1881) [as *Himerometra magnipinna* Clark, 1908]	H	ec	ID	2	[33]
*Pseudanthessius rostellatus* Humes, Ho, 1970	*Phanogenia distincta* (Carpenter, 1888) [as *Comaster distinctus* (Carpenter, 1888)]	Com	ec	MG	47	[75]
Rhynchomolgidae						
*Critomolgus fishelsoni* (Stock, 1967)	*Oligometra serripinna* (Carpenter, 1811)	Col	ec	IL	20	[73]
*Doridicola patulus* (Humes, 1959)	*Cenometra emendatrix* (Bell, 1892)	Col	ec	MG	20	[76]
*Doridicola venustus* (Humes, 1958)	*Cenometra emendatrix* (Bell, 1892)	Col	ec	MG	20	[76]
Synapticolidae						
*Scambicornus pillaii* Stock, 1983 (Figure 2g)	*Capillaster multiradiatus* (Linnaeus, 1758) [as *Capillaster multiradiata* (Linnaeus, 1758)]	Com	ec	IL	1	[72]
Siphonostomatoida						
Asterocheridae						
*Asterocheres crinoidicola* Humes, 2000 (Figure 2h)	Comatulida		ec	JM		[68]
*Asterocheres crinoidicola* Humes, 2000	*Davidaster rubiginosus* (Pourtalès, 1869)	Com	ec	BZ	12.2	[10]
*Asterocheres crinoidicola* Humes, 2000	*Nemaster grandis* Clark, 1909	Com	ec	BZ	32.2	[10]
*Asterocheres spinopaulus* Johnsson, 1998	Comatulida		ec	BR		[67]
*Collocheres amicus* Kim, 2007	*Comanthus briareus* (Bell, 1882) [as *Comantheria rotula* Clark, 1912]	Com	ec	ID	17	[32]
*Collocheres brevipes* Shin, Kim, 2004 (Figure 2i)	*Anneissia solaster* (Clark, 1907) [as *Comanthus solaster* Clark, 1907]	Com	ec	KP	25	[66]
*Collocheres comanthiphilus* Humes, 1987	*Comanthus parvicirrus* (Müller, 1841)	Com	ec	NC	1.5, 5	[22]
*Collocheres comanthiphilus* Humes, 1987	*Comanthus* sp.	Com	ec	NC	1, 3	[22]
*Collocheres comanthiphilus* Humes, 1987	*Comanthus wahlbergii* (Müller, 1843)	Com	ec	ID, NC	0.5, 2, 25	[22]
*Collocheres comanthiphilus* Humes, 1987	*Oxycomanthus bennetti* (Müller, 1841) [as *Comanthus bennetti* (Müller, 1841)]	Com	ec	AU, ID, PH	2, 3, 4, 12, 40	[22]
*Collocheres humesi* Kim, 2007	*Comanthus briareus* (Bell, 1882) [as *Comantheria rotula* Clark, 1912]	Com	ec	ID	17	[32]
*Collocheres inaequalis* Ho, 1982	*Anneissia japonica* (Müller, 1841) [as *Comanthus japonica* (Müller, 1841), *Comanthus japonicus* (Müller, 1841)]	Com	ec	JP		[63]
*Collocheres inflatiseta* Humes, 1987	*Phanogenia multibrachiata* (Carpenter, 1888) [as *Comaster multibrachiatus* (Carpenter, 1888)]	Com	ec	ID	10	[22]
*Collocheres marginatus* Humes, 1987	*Comaster multifidus* (Müller, 1841) [as *Comanthina variabilis* (Bell, 1882)]	Com	ec	AU	9	[22]
*Collocheres parvus* Humes, 1987	*Davidaster rubiginosus* (Pourtalès, 1869)	Com	ec	ID	10	[22]
*Collocheres prionotus* Humes, 1990	*Nemaster grandis* Clark, 1909	Com	ec	ID, MG	0.5, 1	[56]
*Collocheres serrulatus* Humes, 1987	Comatulida	Com	ec	ID	10	[22]
*Collocheres solidus* Shin, Kim, 2004	*Comanthus briareus* (Bell, 1882) [as *Comantheria rotula* Clark, 1912]	Com	ec	KP	25	[66]
*Collocheres solidus* Shin, Kim, 2004	*Anneissia solaster* (Clark, 1907) [as *Comanthus solaster* Clark, 1907]	Com	ec	KP	25	[66]
*Collocheres tamladus* Shin, Kim, 2004	*Comanthus parvicirrus* (Müller, 1841)	Z	ec	KP		[66]
*Collocheres thysanotus* Humes, 1987	*Comanthus* sp.	Com	ec	AU	9	[22]
*Collocheres thysanotus* Humes, 1987	*Comanthus wahlbergii* (Müller, 1843)	Com	ec	AU	9	[22]
*Collocheres titillator* Humes, 1987	*Oxycomanthus bennetti* (Müller, 1841) [as *Comanthus bennetti* (Müller, 1841)]	Com	ec	ID	10	[22]
*Collocheres uncinatus* Stock, 1966	*Comanthus briareus* (Bell, 1882) [as *Comantheria rotula* Clark, 1912]	Col	ec	IL	20	[70]
*Collocheres uncinatus* Stock, 1966	*Anneissia japonica* (Müller, 1841) [as *Comanthus japonica* (Müller, 1841), *Comanthus japonicus* (Müller, 1841)]	Com	ec	ID, MG	0.5, 1, 3	[56]
*Collocheres uncinatus* Stock, 1966	*Phanogenia multibrachiata* (Carpenter, 1888) [as *Comaster multibrachiatus* (Carpenter, 1888)]	H	ec	IL	1, 15	[70]
*Glyptocheres comanthinae* Humes, 1987	*Comaster multifidus* (Müller, 1841) [as *Comanthina variabilis* (Bell, 1882)]	Com	ec	ID	4	[22]
*Glyptocheres extrusus* Humes, 1987	*Davidaster rubiginosus* (Pourtalès, 1869)	Com	ec	NC	1.5	[22]
*Glyptocheres extrusus* Humes, 1987	*Nemaster grandis* Clark, 1909	Com	ec	ID	25	[22]
*Glyptocheres extrusus* Humes, 1987	Comatulida	Com	ec	AU, ID, PH	2, 3, 4, 12, 40	[22]

* Host family abbreviations: A—Antedonidae; Ch—Charitometridae; Col—Colobometridae; Com—Comatulidae; H—Himerometridae; M—Mariametridae; T—Tropiometridae; Z—Zygometridae. ** Symbiosis nature abbreviations: ec—ectosymbiont; en—endosymbiont. *** Country or region abbreviation: AU—Australia; BR—Brazil; BZ—Belize; FR—France; GB—United Kingdom; ID—Indonesia; IE—Ireland; IL—Israel; IN—India; IT—Italy; JM—Jamaica; JP—Japan; KP—Korea; MG—Madagascar; MH—Marshall Islands; NC—New Caledonia; PH—Philippines.
