# Supplementary material for: From Microscale Interactions to Macroscale Patterns in Copepod–Crinoid Symbiosis
Source: Animals (Basel). 2024 Mar 13;14(6):877. doi: 10.3390/ani14060877 (PMC10967334; doi:10.3390/ani14060877)
Supplement: Supplementary file 1 [file animals-14-00877-s001.zip › Table S1.pdf]

Table S1. Crinoids of the World as hosts of copepod crustaceans

Invertebrate Host (valid name and as indicated in the original record)	Copepod species name	Copepod family abbreviation*	Symbiosis nature abbreviation **	Country abbrevatiation ***	Depths	Reference
Comatulida	*Asterocheres crinoidicola* Humes, 2000	A	ec	JM		Kim, 2010
	Asterocheres spinopaulus Johnsson, 1998	A	ec	BR		Johnsson, 2002
	Pseudanthessius madrasensis Reddiah, 1968	P	ec	IN		Reddiah, 1968
Antedonidae						
Antedon bifida (Pennant, 1777)	*Enterognathus comatulae* Giesbrecht, 1900	E	en	GB, IE		Grainger, 1950
Antedon mediterranea (Lamarck, 1816)	*Enterognathus comatulae* Giesbrecht, 1900	E	en	FR, IT		Changeux, Delamare Deboutteville, 1956, Giesbrecht, 1900, Stock, 1959
Charitometridae						
Glyptometra crassa (Clark, 1912)	*Parenterognathus troglodytes* Ohtsuka, Kitazawa, Boxshall, 2010	E	en	JP	775, 780.8 - 781.1	Ohtsuka, Kitazawa, Boxshall, 2010
Colobometridae						

Cenometra emendatrix (Bell, 1892)	*Doridicola patulus* (Humes, 1959)	R	ec	MG	20	Humes, Stock, 1973
Cenometra emendatrix (Bell, 1892)	*Doridicola venustus* (Humes, 1958)	R	ec	MG	20	Humes, Stock, 1973
Cenometra emendatrix (Bell, 1892)	*Pseudanthessius major* Stock, 1967	P	ec	MG	10, 20	Stock, 1967
Decametra chadwicki (Clark, 1911)	*Entherognathus lateripes* Stock, 1966	E	en	IL	20	Stock, 1966
Oligometra serripinna (Carpenter, 1811)	*Collocheres uncinatus* Stock, 1966	A	ec	IL	20	Stock, 1966
Oligometra serripinna (Carpenter, 1811)	*Critomolgus fishelsoni* (Stock, 1967)	R	ec	IL	20	Stock, 1967
Oligometra serripinna (Carpenter, 1811)	*Entherognathus lateripes* Stock, 1966	E	en	IL	20	Stock, 1966
Comasteridae						
Anneissia bennetti (Müller, 1841)	*Pseudanthessius comanthi* Humes, 1972	P	ec	MH	4, 8	Humes, 1972
Anneissia japonica (Müller, 1841)	*Collocheres inaequalis* Ho, 1982	A	ec	JP		Ho, 1982
Anneissia japonica (Müller, 1841)	*Collocheres solidus* Shin, Kim, 2004	A	ec	KP	25	Shin, Kim, 2004
Anneissia solaster (Clark, 1907)	*Collocheres brevipes* Shin, Kim, 2004	A	ec	KP	25, 40	Shin, Kim, 2004
Anneissia solaster (Clark, 1907)	*Collocheres solidus* Shin, Kim, 2004	A	ec	KP	25, 40	Shin, Kim, 2004

Capillaster multiradiatus (Linnaeus, 1758)	*Collocheres prionotus* Humes, 1990	A	ec	ID, MG	0.5, 1	Humes, 1990
Capillaster multiradiatus (Linnaeus, 1758)	*Collocheres uncinatus* Stock, 1966	A	ec	ID, MG	0.5, 1, 3	Humes, 1990
Capillaster multiradiatus (Linnaeus, 1758)	*Scambicornus pillaii* Stock, 1983	S	ec	IL	1	Stock, 1983
Comanthus sp.	*Collocheres comanthiphilus* Humes, 1987	A	ec	NC	1, 3	Humes, 1987
Comanthus briareus (Bell, 1882)	*Collocheres amicus* Kim, 2007	A	ec	ID	17	Kim, 2007
Comanthus briareus (Bell, 1882)	*Collocheres humesi* Kim, 2007	A	ec	ID	17	Kim, 2007
Comanthus briareus (Bell, 1882)	*Collocheres serrulatus* Humes, 1987	A	ec	ID	10	Humes, 1987
Comanthus briareus (Bell, 1882)	*Collocheres thysanotus* Humes, 1987	A	ec	AU	9	Humes, 1987
Comanthus parvicirrus (Müller, 1841)	*Collocheres comanthiphilus* Humes, 1987	A	ec	NC	1.5, 5	Humes, 1987
Comanthus parvicirrus (Müller, 1841)	*Glyptocheres extrusus* Humes, 1987	A	ec	NC	1.5	Humes, 1987
Comanthus wahlbergii (Müller, 1843)	*Collocheres comanthiphilus* Humes, 1987	A	ec	ID, NC	0.5, 2, 25	Humes, 1987
Comanthus wahlbergii (Müller, 1843)	*Glyptocheres extrusus* Humes, 1987	A	ec	ID	25	Humes, 1987

Comanthus wahlbergii (Müller, 1843)	*Pseudanthessius comanthi* Humes, 1972	P	ec	ID	25	Humes, 1987
Comaster multifidus (Müller, 1841)	*Collocheres marginatus* Humes, 1987	A	ec	AU	9	Humes, 1987
Comaster multifidus (Müller, 1841)	*Collocheres thysanotus* Humes, 1987	A	ec	AU	9	Humes, 1987
Comaster schlegeli (Carpenter, 1881)	*Glyptocheres comanthinae* Humes, 1987	A	ec	ID	4	Humes, 1987
Davidaster rubiginosus (Pourtalès, 1869)	*Asterocheres crinoidicola* Humes, 2000	A	ec	BZ	12.2	Humes, 2000
Nemaster grandis Clark, 1909	*Asterocheres crinoidicola* Humes, 2000	A	ec	BZ	32.2	Humes, 2000
Oxycomanthus bennetti (Müller, 1841) (= *Comanthus bennetti* (Müller, 1841))	*Collocheres comanthiphilus* Humes, 1987	A	ec	AU, ID, PH	2, 3, 4, 12, 40	Humes, 1987
Oxycomanthus bennetti (Müller, 1841) (= *Comanthus bennetti* (Müller, 1841))	*Glyptocheres extrusus* Humes, 1987	A	ec	AU, ID, PH	2, 3, 4, 12, 40	Humes, 1987
Oxycomanthus bennetti (Müller, 1841) (= *Comanthus bennetti* (Müller, 1841))	*Pseudanthessius comanthi* Humes, 1972	P	ec	AU, ID, PH	2, 3, 4, 10, 12, 40	Humes, 1987
Phanogenia distincta (Carpenter, 1888) (= *Comaster distinctus* (Carpenter, 1888))	*Pseudanthessius rostellatus* Humes, Ho, 1970	P	ec	MG	47	Humes, Ho, 1970
Phanogenia gracilis (Hartlaub, 1893) (= *Comaster gracilis* (Hartlaub, 1893))	*Collocheres titillator* Humes, 1987	A	ec	ID	10	Humes, 1987

Phanogenia multibrachiata (Carpenter, 1888) (= *Comaster multibrachiatus* (Carpenter, 1888))	*Collocheres inflatiseta* Humes, 1987	A	ec	ID	10	Humes, 1987
Phanogenia multibrachiata (Carpenter, 1888) (= *Comaster multibrachiatus* (Carpenter, 1888))	*Collocheres parvus* Humes, 1987	A	ec	ID	10	Humes, 1987
Himerometridae						
Heterometra africana (Clark, 1911)	*Pseudanthessius major* Stock, 1967	P	ec	MG	17, 18, 25, 29, 34	Stock, 1967
Heterometra africana (Clark, 1911)	*Pseudanthessius minor* Stock, 1967	P	ec	MG	18	Stock, 1967
Heterometra savignii (Müller, 1841) (= *Heterometra savignyi* (Müller, 1841))	*Collocheres uncinatus* Stock, 1966	A	ec	IL	1, 15	Stock, 1966
Heterometra savignii (Müller, 1841) (= *Heterometra savignyi* (Müller, 1841))	*Entherognathus lateripes* Stock, 1966	E	en	IL	10	Stock, 1966
Heterometra savignii (Müller, 1841) (= *Heterometra savignyi* (Müller, 1841))	*Kelleria gradata* Stock, 1967	K	ec	IL	15	Stock, 1967
Heterometra savignii (Müller, 1841) (= *Heterometra savignyi* (Müller, 1841))	*Pseudanthessius major* Stock, 1967	P	ec	IL	10, 15	Stock, 1967
Himerometra robustipinna (Carpenter, 1881)	*Pseudanthessius major* Stock, 1967	P	ec	NC	1	Humes, 1977
Himerometra robustipinna (Carpenter, 1881)	*Pseudanthessius planus* Kim, 2007	P	ec	ID	2	Kim, 2007
Mariametridae						
Dichrometra flagellata (Müller, 1841)	*Pseudanthessius angularis* Humes, Ho, 1970	P	ec	MG	1	Humes, Ho, 1970
Dichrometra flagellata (Müller, 1841)	*Pseudanthessius major* Stock, 1967	P	ec	MG	1, 2, 6	Stock, 1967

Dichrometra flagellata (Müller, 1841)	*Pseudanthessius minor* Stock, 1967	P	ec	MG	2	Stock, 1967
Lamprometra palmata (Müller, 1841) (= *Lamprometra klunzingeri* (Hartlaub, 1890))	*Pseudanthessius major* Stock, 1967	P	ec	MG	1, 13	Stock, 1967
Lamprometra palmata (Müller, 1841) (= *Lamprometra klunzingeri* (Hartlaub, 1890))	*Pseudanthessius minor* Stock, 1967	P	ec	IL, MG	0.5, 13	Stock, 1967
Lamprometra sp.	*Enterognathus inabai* Ohtsuka, Shimomura, Kitazawa, 2012	E	en	JP	46.7	Ohtsuka, Shimomura, Kitazawa, 2012
Liparometra sp.	*Pseudanthessius minor* Stock, 1967	P	ec	MG	15, 23, 35	Stock, 1967
Liparometra sp.	*Pseudanthessius angularis* Humes, Ho, 1970	P	ec	MG	15, 23, 35	Stock, 1967
Stephanometra indica (Smith, 1876) (= *Stephanometra spicata* (Carpenter, 1881))	*Pseudanthessius major* Stock, 1967	P	ec	MG	2, 6	Humes, Ho, 1970
Stephanometra indica (Smith, 1876) (= *Stephanometra spicata* (Carpenter, 1881))	*Pseudanthessius major* Stock, 1967	P	ec	MG	2, 13, 17	Stock, 1967
Stephanometra indica (Smith, 1876) (= *Stephanometra spicata* (Carpenter, 1881))	*Pseudanthessius major* Stock, 1967	P	ec	NC	3	Humes, 1977
Tropiometridae						
Tropiometra afra (Hartlaub, 1890)	*Pseudanthessius madrasensis* Reddiah, 1968	P	ec	NC	1.5, 2, 3	Humes, 1977
Tropiometra carinata (Lamarck, 1816)	*Pseudanthessius madrasensis* Reddiah, 1968	P	ec	MG	0.5, 1, 1.5, 2, 3, 15	Humes, Ho, 1970
Zygometridae						

Catoptometra rubroflava (Clark, 1908)	*Collocheres tamladus* Shin, Kim, 2004	A	ec	KP	Shin, Kim, 2004

* Copepod family Abbreviation: A – Asterocheridae, E – Enterognathidae, K – Kelleriidae, P – Pseudanthessiidae, R – Rhynchomolgidae, S – Synapticolidae.

** Symbiosis nature abbreviation: ec – ectosymbiont, en – endosymbiont.

*** Country abbreviation: AU – Australia, BR – Brazil, BZ – Belize, FR – France, GB – United Kingdom, ID – Indonesia, IE – Ireland, IL – Israel, IN – India, IT – Italy, JM – Jamaica, JP – Japan, KP – Korea, MG – Madagascar, MH – Marshall Islands, NC – New Caledonia, PH – Philippines.
