# Supplementary material for: From Microscale Interactions to Macroscale Patterns in Copepod–Crinoid Symbiosis
Source: Animals (Basel). 2024 Mar 13;14(6):877. doi: 10.3390/ani14060877 (PMC10967334; doi:10.3390/ani14060877)
Supplement: Supplementary file 1 [file animals-14-00877-s001.zip › Table S2.pdf]

Table S2. Description of the dataset with specific information relative to column names, description, units, and attribute type.

Attribute	Column_name	Description	Units	Attribute_Type
Record number	rID	Unique number corresponding to specific occurrence		Integer
Record ID	recordID	A structured code incorporating a concise article reference, region and country observation identifiers, shorthand for the location coordinates, and specific abbreviations for the symbiont and host families, complemented by a distinct number.		Text
Aphia ID of symbiont	aphiaID_Symbiont	Unique number for taxon from WoRMS database		Integer
Kingdom of symbiont	kingdom_Symbiont	Taxonomic rank below Domain		Text
Phylum of symbiont	phylum_Symbiont	Taxonomic rank below Kingdom		Text
Class of symbiont	class_Symbiont	Taxonomic rank below Phylum		Text
Order of symbiont	order_Symbiont	Taxonomic rank below Class		Text
Family of symbiont	family_Symbiont	Taxonomic rank below Order		Text
Genus of symbiont	genus_Symbiont	Taxonomic rank below Family and first element in the Latin binomial name		Text
Specific epithet of symbiont	specificEpithet_Symbiont	Second element in the Latin binomial name		Text
Scientific name authorship of symbiont	scientificNameAuthorship_Symbiont	The name of the individual author or team responsible for the species' discovery.		Text
Symbiont ID	symbiontID	Reviewed species name		Text
Taxon rank of symbiont	taxonRank_Symbiont	Taxonomic rank information (e.g., genus, species)		Text
Taxonomic status of symbiont	taxonomicStatus_Symbiont	Taxonomic status information (e.g., accepted, unaccepted)		Text
Link of symbiont	link_Symbiont	Link to taxon in WoRMS database		Text
Female Body Length	femaleLength	Length of the female specimen	μm	Text
Female Body Weight	femaleWidth	Width of the female specimen	μm	Text
Male Body Length	maleLength	Length of the male specimen	μm	Text
Male Body Weight	maleWidth	Width of the male specimen	μm	Text
Aphia ID of host	aphiaID_Host	Unique number for taxon from WoRMS database		Integer
Kingdom of host	kingdom_Host	Taxonomic rank below Domain		Text
Phylum of host	phylum_Host	Taxonomic rank below Kingdom		Text
Class of host	class_Host	Taxonomic rank below Phylum		Text
Order of host	order_Host	Taxonomic rank below Class		Text
Family of host	family_Host	Taxonomic rank below Order		Text
Genus of host	genus_Host	Taxonomic rank below Family and first element in the Latin binomial name		Text
Specific epithet of host	specificEpithet_Host	Second element in the Latin binomial name		Text
Scientific name authorship of host	scientificNameAuthorship_Host	The name of the individual author or team responsible for the species' discovery.		Text
Host ID	hostID	Reviewed species name		Text
Taxon rank of host	taxonRank_Host	Taxonomic rank information (e.g., genus, species)		Text
Taxonomic status of host	taxonomicStatus_Host	Taxonomic status information (e.g., accepted, unaccepted)		Text
Link of host	link_Host	Link to taxon in WoRMS database		Text
Site ID	siteID	Unique number for locality		Text
Region code	regionCode	Unique number for region		Text
Region	region	Division of the World Ocean (Spalding et al., 2007)		Text

Ocean	ocean	The name of the ocean in which the locality occurs.		Text
Water body	waterBody	The name of the water body in which the locality occurs.		Text
Island	island	The name of the island near which the locality occurs.		Text
Country	country	The name of the country in which the locality occurs.		Text
Country code	countryCode	The standard code (ISO 3166-1-alpha-2) for the country in which the locality occurs.		Text
Locality	locality	Area where the taxon was found		Text
Exact Location Description	verbatimLocalition	A comprehensive description of the location from the original article		Text
Geocoordinates	geocoordinates	A combined representation of both latitude and longitude	Degrees Minutes Seconds (DMS)	Text
Latitude	latitude	Coordinate that specifies the N–S position of a point on the Earth surface	Degrees Minutes Seconds (DMS)	Text
Longitude	longitude	Coordinate that specifies the E–W position of a point on the Earth surface	Degrees Minutes Seconds (DMS)	Text
Decimal geocoordinates	decimalGeocoordinates	A combined representation of both latitude and longitude	Decimal degrees, WGS84	Numeric
Decimal latitude	decimalLatitude	Coordinate that specifies the N–S position of a point on the Earth surface	Decimal degrees, WGS84	Numeric
Decimal longitude	decimalLongitude	Coordinate that specifies the E–W position of a point on the Earth surface	Decimal degrees, WGS84	Numeric
Coordinate uncertainty	coordinateUncertaintyInMeters	The horizontal distance from the given decimal latitude and longitude describing the smallest circle containing the whole of the location.	m	Integer
Minimum depth	minimumDepthInMeters	The vertical distance below sea level	m	Integer
Maximum depth	maximumDepthInMeters	The vertical distance below sea level	m	Integer
Collecting method	collectingMethod	The method of taking the sample		Text
Finding method	findingMethod	The method of finding copepods in the sample		Text
Type of association	note	Describes the nature of the interaction.		Text
Host interaction site	locationAtHost	The general location or site on the host where the copepod interacts or resides.		Text
Event date	eventDate	Date of sampling.		Date
Year	year	The four-digit year of the record. Format: yyyy.		Integer
Month	month	The ordinal month of the occurrence record. Format: mm.		Integer
Article ID	articleID	Short reference		Text
Reference	reference	Full reference to article		Text
